# Enhancing soil health and fruit yield through *Tephrosia* biomass mulching in rainfed guava (*Psidium guajava* L.) orchards

**DOI:** 10.1038/s41598-024-64814-x

**Published:** 2024-06-17

**Authors:** Abeer Ali, Bikash Das, M. K. Dhakar, S. K. Naik, V. B. Patel, G. P. Mishra, P. K. Sarkar, Reshma Shinde, A. K. Jha, B. P. Bhatt

**Affiliations:** 1 ICAR-Indian Agricultural Research Institute, Barhi, Hazaribagh, Jharkhand 825405 India; 2 The Graduate School, Indian Agricultural Research Institute, New Delhi, 110012 India; 3 Farming System Research Centre for Hill and Plateau Region, ICAR Research Complex for Eastern Region, Plandu, Ranchi, Jharkhand 834010 India; 4 ICAR-National Research Centre on Litchi, Muzaffarpur, Bihar 842002 India; 5 Horticultural Science Division, Krishi Anusandhan Bhawan - II, ICAR, New Delhi, 110012 India; 6 Division of Seed Science and Technology, ICAR-Indian Agricultural Research Institute, New Delhi, 110012 India; 7 ICAR Research Complex for NEH Region, Tripura Centre, Lembucherra, West Tripura 799210 India; 8 Natural Resource Management Division, Krishi Anusandhan Bhawan - II, ICAR, New Delhi, 110012 India

**Keywords:** Carbon sequestration, Nutrients, Sustainability, Low fertility, Litters decomposition and *Tephrosia candida*, Plant sciences, Climate sciences

## Abstract

Leguminous crop *Tephrosia candida* has high biomass production and contains a substantial quantity of nutrients within its biomass. Starting in 2019, a long-term study was done to find the best *Tephrosia candida* dose for mulching in guava orchards. The study had four treatments: T_1_ = 3.0 kg dry biomass m^−2^ of the plant basin, T_2_ = 2.0 kg, T_3_ = 1.0 kg, and T_4_ = control (no mulch). Every year, the treatments imposed in the month of August. The third year (2021–2022) results indicated that mulching with 3 kg of biomass m^−2^ increased trunk diameter, fruit yield, fruit weight, specific leaf area, total leaf chlorophyll, and leaf macro- and micro-nutrients. At 3.0 kg m^−2^, mulching improved soil properties such as EC, available nitrogen, available phosphorus, exchangeable potassium, DTPA extractable micronutrients (Fe, Zn, Cu, and Mn), total organic carbon (C_toc_), soil organic carbon (C_soc_), organic carbon fractions, and microbial biomass carbon between 0–0.15 m and 0.15–0.30 m. There was an increasing trend in dehydrogenase activity (DHA) and fluorescein diacetate (FDA). The *Tephrosia* leaf litter exhibited decay constants of 1.27 year^−1^, and the carbon content was 40.11%. Therefore, applying *Tephrosia* biomass mulching at a rate of 3.0 kg m^−2^ is a viable long-term solution for enhancing soil fertility and sequestering carbon.

## Introduction

Guava (*Psidium guajava* L.) is an important fruit crop in tropical and subtropical regions of India. It is adaptable to various soil and climatic conditions, including clay, sand, gravel bars, limestone, and slightly acidic to alkaline soils (pH ranging from 5.5 to 7.5)^1^. Guava responds well to proper manuring and fertilization to achieve optimal growth and yield. The Eastern Plateau and Hill Region (EPHR) of India has been a traditional guava growing region in the country. However, guava orchards in this region face challenges due to poor soil fertility, low organic carbon content, limited water holding capacity, and nutrient deficiencies, resulting in lower yields as compared to other guava-growing regions^2^. Improving soil organic carbon can be the most important step towards ameliorating the edaphic constraints under the EPHR. Although, application of farmyard manure is the most effective means for improving the soil organic carbon, its limited availability in the region combined with its higher cost leads to its minimal application by the farmers, particularly in fruit orchards.

The present scenario demands an economically feasible and environment friendly approach, particularly for the rainfed upland. Enriching the plant basins with leafy biomass is an effective way to improve soil organic matter and nutrient content. While green manuring with crops like *sesbania* or *crotalaria* is effective for plant-based organic carbon management, their annual nature and need for timely incorporation limit their widespread adoption in fruit production systems. To overcome this, integrating biomass-yielding perennial plants has proven successful in improving soil fertility. A number of perennial biomass-yielding plants are being used in agroforestry systems worldwide to improve soil fertility through nutrient recycling. Subabul (*Leucaena leucocephala*) is the most commonly used plant for this purpose^3^. However, difficulties in its eradication and high seed dispersal rate hinder its integration into fruit based production systems in the region. On the other hand, *Tephrosia candida,* a perennial leguminous plant, has shown promising results in improving soil fertility when integrated into Bael (*Aegle marmelos* Corr.) based production systems under EPHR due to its higher biomass yield and shorter lifespan of 5–6 years^3^. *Tephrosia candida* (Roxb) is native to the Himalayan tropical foothills of India and is naturalized in Southeast Asia. The species is promising because of its higher biomass yield^4^, ability to reduce soil acidity due to its higher amounts of calcium and magnesium^5^. The plant thrives in drought conditions, grows swiftly, and poses little threat to animals. It requires no maintenance, making it an ideal material for biomass mulching. According to Das^6^, *Tephrosia candida* leaves have nitrogen, phosphorus, potassium, zinc, and copper contents of 2.94%, 0.16%, 1.06%, 35.35 ppm and 19.18 ppm, respectively and mulching of *Tephrosia candida* in the bael resulted in significant improvements in soil fertility and plant growth parameters.

Several fruit crops have shown positive outcomes from the use of biomass mulching. The beneficial effects have been recorded mainly on soil physical properties, viz. water holding capacity^2^, bulk density^7^, chemical properties, viz. organic carbon^6^, available nutrients^6^ and biological properties viz. microbial biomass^8^, enzymatic activities^9^. etc. However, the effects vary with different mulching material and their interactions with soil and plants. Similarly, the amount of biomass to apply as mulch can also impact the outcomes of biomass mulching. Apart from the beneficial effects, excessive application of biomass in the plant basin may also have detrimental effects on the fruit plants, including nitrogen immobilisation. Keeping in view the absence of literature on *Tephrosia* mulching in guava, it was necessary to standardize the quantity of *Tephrosia* biomass to be applied as mulch in guava orchards for widespread adoption of this practice. Therefore, we investigated to measure the impact of various Tephrosia biomass mulching doses on the physical, chemical, and biological properties of the soil, as well as the fruit yield of guava plants grown under rainfed conditions.

## Results

### Soil moisture dynamics

**Figure 1:**
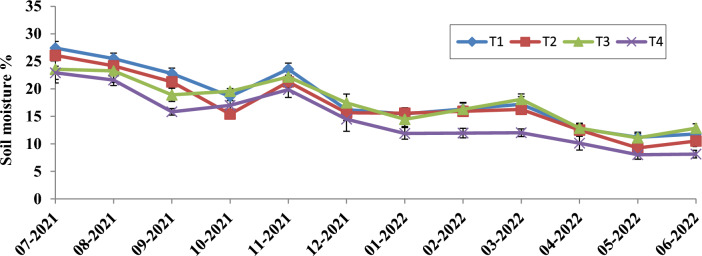
Effect of biomass mulching of *Tephrosia* on soil moisture (%) at 0–15 cm depth. The error bars shows the standerd error.

**Figure 2:**
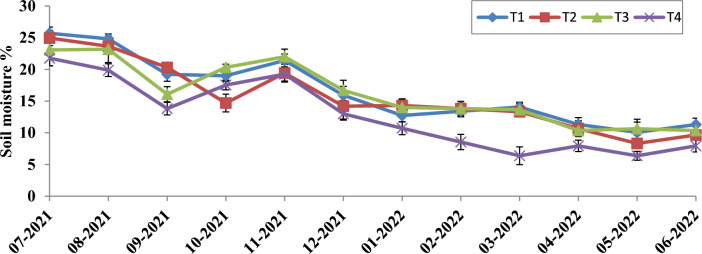
Effect of biomass mulching of *Tephrosia* on soil moisture % at 15–30 cm depth. The error bars shows the standerd error.

Soil water content varied significantly from July 2021 to June 2022 at 0–30 cm. The soils with 3.0 kg m^−2^ biomass mulch had significantly higher soil *moisture* content even in the dryer months (Figs. 1 and 2). *The treatment resulted in* 36.95% and 27.77% higher moisture content in the 0–15 cm and 15–30 cm soil depths, respectively, over the non-mulched soil.

### Soil electrical conductivity and nutrients

Significant variation (*P* ≤ 0.05) in soil electrical conductivity and soil nutrients (available nitrogen, available phosphorus, exchangeable potassium and DTPA extractable Mn, Zn, Fe and Cu) were observed among treatments at both soil depths (Tables 1 and 2). As compared to control, mulching at 3.0 kg m^−2^ significantly increased soil EC by 150% and 22.22%, available nitrogen by 23.02% and 23.32%, and available phosphorus by 92.77% and 101.03% at 0–15 cm and 15–30 cm soil depths, respectively. *Tephrosia* mulching increased exchangeable potassium by 47.47% (0–15 cm) and 60.39% (15–30 cm) as compared to control. In the case of DTPA extractable Mn, Zn, Fe and Cu, the highest values were recorded under mulching dose of 3.0 kg m^−2^.

**Table 1:** Effect of *Tephrosia* biomass mulching on soil EC and content of available nitrogen, phosphorus and exchangeable potassium (mean ± standard error).

Treatments	Soil EC (mS cm^−1^)		Available nitrogen (kg ha^−1^)		Available phosphorus (kg ha^−1^)		Exchangeable potassium (kg ha^−1^)	
	0–15 cm	15–30 cm	0–15 cm	15–30 cm	0–15 cm	15–30 cm	0–15 cm	15–30 cm
T_1_ (3.0 kg dry biomass m^−2^)	0.25 ± 0.01^a^	0.11 ± 0.00^a^	246.96 ± 3.53^a^	245.04 ± 3.47^a^	33.85 ± 1.77^a^	25.39 ± 1.79^a^	372.19 ± 19.57^a^	323.63 ± 13.35^a^
T_2_ (2.0 kg dry biomass m^−2^)	0.16 ± 0.02^b^	0.10 ± 0.00^b^	226.59 ± 9.78^ab^	226.06 ± 8.40^ab^	27.05 ± 1.43^b^	20.77 ± 1.74^a^	293.53 ± 15.55^b^	235.11 ± 21.45^b^
T_3_ (1.0 kg dry biomass m^−2^)	0.15 ± 0.00^b^	0.11 ± 0.00^b^	226.33 ± 10.38^ab^	224.45 ± 10.38^ab^	24.64 ± 1.30^b^	22.93 ± 1.29^a^	256.25 ± 2.28^b^	248.94 ± 5.67^c^
T_4_ (No mulching)	0.10 ± 0.00^c^	0.09 ± 0.00^c^	200.74 ± 13.23^b^	198.70 ± 13.43^b^	17.56 ± 2.08^c^	12.70 ± 1.19^b^	252.38 ± 6.04^b^	201.77 ± 4.20^c^

The value indicated with in the column by the same letters are not significantly different at 0.05 level of probability by DMRT.

**Table 2:** Effect of *Tephrosia* biomass mulching on soil available Mn, Fe, Zn and Cu (mean ± standard error).

Treatments	DTPA extractable Mn (ppm)		DTPA extractable Fe (ppm)		DTPA extractable Zn (ppm)		DTPA extractable Cu (ppm)	
	0–15 cm	15–30 cm	0–15 cm	15–30 cm	0–15 cm	15–30 cm	0–15 cm	15–30 cm
T_1_ (3.0 kg dry biomass m^−2^)	123.34 ± 3.63^a^	117.85 ± 4.63^a^	117.20 ± 3.21^a^	109.67 ± 4.01^a^	2.35 ± 0.14^a^	2.03 ± 0.16^a^	1.36 ± 0.15^a^	1.25 ± 0.12^a^
T_2_ (2.0 kg dry biomass m^−2^)	102.00 ± 4.19^b^	103.88 ± 4.63^ab^	111.30 ± 3.83^b^	109.09 ± 3.86^a^	2.24 ± 0.10^a^	2.04 ± 0.09^a^	0.95 ± 0.02^b^	0.88 ± 0.04^b^
T_3_ (1.0 kg dry biomass m^−2^)	108.28 ± 8.24^bc^	99.72 ± 3.19^b^	100.98 ± 3.21^bc^	99.00 ± 3.20^b^	2.20 ± 0.18^a^	1.86 ± 0.18^a^	0.77 ± 0.06^bc^	0.74 ± 0.04^bc^
T_4_ (No mulching)	90.46 ± 2.63^c^	90.23 ± 2.26^c^	87.30 ± 1.61^c^	85.30 ± 1.69^c^	1.35 ± 0.06^b^	1.12 ± 0.05^b^	0.63 ± 0.03^c^	0.59 ± 0.03^c^

The values indicated with in the column by the same letters are not significantly different at 0.05 level of probability by DMRT.

### Soil organic carbon fractions

Significant variation (*P* ≤ 0.05) in total organic carbon (C_toc_), oxidizable organic carbon (C_soc_) and fractions (C_*frac*1_, C_*frac*2_ and C_*frac*4_) was observed among treatments throughout soil depths (0–30 cm) (Tables 3, 4 and 5). C_toc_, C_soc_, C_*frac*1_ and C_*frac*2_ decreased with the increasing depth of the soil. The control recorded the lowest C_toc_ at both soil depths. The maximum C_toc_ was observed with the application of 3.0 kg *Tephrosia* biomass, while the application of 2.0 kg and 1.0 kg *Tephrosia* were found to be at par with each other. The C_soc_ varied from 24.14 to 30.85 Mg C/ha among the different treatments under study. There was 27.79% increment in oxidizable organic carbon by the application of *Tephrosia* biomass mulch @ 3.0 kg m^−2^. The carbon fractions varied in the order of C_*frac*1_ > C_*frac*2_ > C_*frac*4_ > C_*frac*3_, with greater values at 0–15 cm.

**Table 3:** Effect of *Tephrosia* biomass mulching on soil oxidizable and total organic carbon fractions of guava orchard (mean ± standard error).

Treatments	Oxidizable organic carbon (Mg ha^−1^) (Csoc)			Total organic carbon (Mg ha^−1^) (C_toc_)		
	0–15 cm	15–30 cm	Total	0–15 cm	15–30 cm	Total
T_1_ (3.0 kg dry biomass m^−2^)	16.58 ± 0.25^a^	14.26 ± 0.26^a^	30.85 ± 0.47^a^	21.73 ± 0.51^a^	18.51 ± 0.43^a^	40.25 ± 0.48^a^
T_2_ (2.0 kg dry biomass m^−2^)	15.68 ± 0.26^a^	13.68 ± 0.43^ab^	29.37 ± 0.61^ab^	19.94 ± 0.19^b^	16.98 ± 0.37^b^	36.92 ± 0.47^b^
T_3_ (1.0 kg dry biomass m^−2^)	15.49 ± 0.39^b^	12.85 ± 0.31^b^	28.35 ± 0.68^b^	19.23 ± 0.36^bc^	16.24 ± 0.41^b^	35.47 ± 0.49^b^
T_4_ (No mulching)	13.61 ± 0.42^c^	10.53 ± 0.21^c^	24.14 ± 0.43^c^	17.91 ± 0.45^c^	12.32 ± 0.32^c^	30.23 ± 0.49^c^

The values indicated with in the column by the same letters are not significantly different at 0.05 level of probability by DMRT.

**Table 4:** Effect of *Tephrosia* biomass mulching on very labile and labile soil organic carbon fractions of guava orchard (mean ± standard error).

Treatments	Very labile (Mg ha^−1^) (C_*frac*1_)			Labile (Mg ha^−1^) (C_*frac*2_)		
	0–15 cm	15–30 cm	Total	0–15 cm	15–30 cm	Total
T_1_ (3.0 kg dry biomass m^−2^)	8.06 ± 0.24^a^	6.85 ± 0.22^a^	14.91 ± 0.36^a^	5.57 ± 0.23^a^	4.71 ± 0.41^a^	10.28 ± 0.59^a^
T_2_ (2.0 kg dry biomass m^−2^)	7.48 ± 0.24^a^	6.69 ± 0.19^a^	14.17 ± 0.31^ab^	5.19 ± 0.20^a^	4.00 ± 0.28^ab^	9.19 ± 0.45^ab^
T_3_ (1.0 kg dry biomass m^−2^)	7.31 ± 0.47^a^	6.17 ± 0.23^a^	13.49 ± 0.34^b^	4.82 ± 0.15^a^	3.54 ± 0.28^bc^	8.37 ± 0.10^bc^
T_4_ (No mulching)	6.29 ± 0.30^b^	4.99 ± 0.23^b^	11.28 ± 0.42^c^	3.98 ± 0.29^b^	3.02 ± 0.09^c^	7.00 ± 0.41^c^

The values indicated with in the column by the same letters are not significantly different at 0.05 level of probability by DMRT.

**Table 5:** Effect of *Tephrosia* biomass mulching on less labile and non-labile soil organic carbon fractions of guava orchard (mean ± standard error).

Treatments	Less labile (Mg ha^−1^) (C_*frac*3_)			Non labile (Mg ha^−1^) (C_*frac*4_)		
	0–15 cm	15–30 cm	Total	0–15 cm	15–30 cm	Total
T_1_ (3.0 kg dry biomass m^−2^)	2.94 ± 0.12	2.70 ± 0.22	5.64 ± 0.32	5.15 ± 0.51	4.24 ± 0.44^a^	9.39 ± 0.47^a^
T_2_ (2.0 kg dry biomass m^−2^)	3.00 ± 0.38	2.99 ± 0.29	5.99 ± 0.52	4.26 ± 0.38	3.29 ± 0.46^a^	7.55 ± 0.79^ab^
T_3_ (1.0 kg dry biomass m^−2^)	3.35 ± 0.43	3.13 ± 0.15	6.48 ± 0.55	3.74 ± 0.37	3.38 ± 0.23^a^	7.12 ± 0.25^b^
T_4_ (No mulching)	3.34 ± 0.40	2.50 ± 0.21	5.85 ± 0.41	4.30 ± 0.82	1.78 ± 0.23^b^	6.08 ± 0.69^b^

The values indicated with in the column by the same letters are not significantly different at 0.05 level of probability by DMRT.

### Soil biological properties

**Figure 3:**
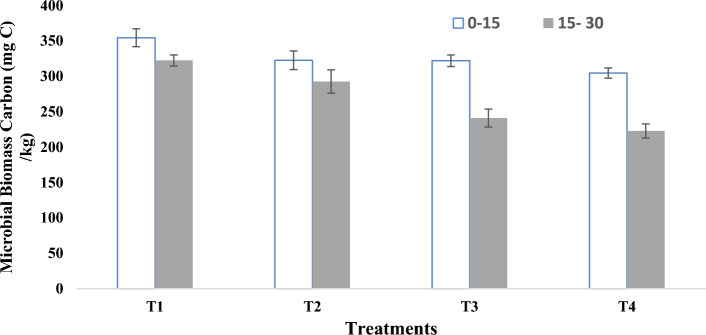
Effect of biomass mulching of *Tephrosia* on soil microbial biomass carbon (mean ± standard error).

Mulching with the biomass of Tephrosia resulted in significant improvements in different soil biological properties over that of control. In the case of microbial biomass carbon (C_mbc_), mulching @ 3.0 kg m^−2^ resulted in 16.39% (0–15 cm) and 44.66% (15–30 cm) higher C_mbc_ than the control (Fig. 3). Significantly higher activities of soil dehydrogenase activity (DHA) and Fluorescein diacetate (FDA) were recorded with the application of *Tephrosia* biomass mulching compared to the control (Tables 6 and 7). In the treatment with 3 kg m^−2^ biomass mulch, DHA ranged from 50.11 to 65.35 µg TPF day^−1^ g soil^−1^ between November 2021 and April 2022. The significantly highest value was recorded in the treatment with 3 kg m^−2^ (65.35 µg TPF day^−1^ g soil^−1^) in April 2022. In April, the mean increase in DHA in the mulched plot was 108% higher than that in the control. A similar trend was recorded in the FDA. The significantly highest value was recorded in April, 2022 (95.16 mg fluorescein kg soil^−1^ h^−1^). The mean FDA in the mulched plot during the month of April was 104.4% higher than that in the control.

**Table 6:** Effect of biomass mulching of *Tephrosia* bio-mulching on soil dehydrogenase activity in guava orchard.

Treatments	Soil dehydrogenase activity (µg TPF day^−1^ g soil^−1^)					
	Nov-21	Dec-21	Jan-22	Feb-22	Mar-22	Apr-22
T_1_ (3.0 kg dry biomass m^−2^)	57.94 ± 2.55^a^	51.76 ± 3.05^a^	50.11 ± 3.28^a^	57.87 ± 1.88^a^	61.78 ± 1.28^a^	65.35 ± 12.76^a^
T_2_ (2.0 kg dry biomass m^−2^)	36.91 ± 3.73^b^	36.38 ± 1.22^b^	45.96 ± 2.79^a^	36.87 ± 1.70^b^	59.15 ± 1.49^a^	55.44 ± 2.49^b^
T_3_ (1.0 kg dry biomass m^−2^)	43.49 ± 2.50^b^	37.35 ± 2.71^b^	43.89 ± 2.33^a^	30.55 ± 1.22^c^	40.84 ± 0.78^b^	29.00 ± 1.55^c^
T_4_ (No mulching)	20.00 ± 2.56^c^	16.94 ± 1.64^c^	19.87 ± 1.92^b^	12.72 ± 0.99^d^	23.68 ± 0.96^c^	24.23 ± 1.82^c^

^#^The values indicated with in the column by the same letters are not significantly different at 0.05 level of probability by DMRT.

**Table 7:** Effect of biomass mulching of *Tephrosia* bio-mulching on soil fluorescein diacetate activity in guava orchard.

Treatments	Fluorescein diacetate (mg fluorescein kg soil^−1^ h^−1^)					
	Nov-21	Dec-21	Jan-22	Feb-22	Mar-22	Apr-22
T_1_ (3.0 kg dry biomass m^−2^)	65.06 ± 5.57^a^	54.83 ± 2.25^a^	52.22 ± 4.21^a^	93.41 ± 3.61^a^	67.35 ± 2.17^a^	95.16 ± 6.15^a^
T_2_ (2.0 kg dry biomass m^−2^)	37.85 ± 2.28^b^	28.30 ± 2.39^b^	34.25 ± 3.85^b^	79.56 ± 5.70^a^	44.85 ± 3.44^b^	63.92 ± 8.42^b^
T_3_ (1.0 kg dry biomass m^−2^)	35.18 ± 3.10^b^	44.52 ± 1.39^c^	54.94 ± 3.18^a^	58.45 ± 5.83^b^	38.03 ± 1.36^b^	49.50 ± 8.31^bc^
T_4_ (No mulching)	23.95 ± 1.71^c^	16.81 ± 2.52^d^	20.94 ± 3.26^c^	21.78 ± 5.29^c^	23.68 ± 3.65^c^	34.00 ± 1.43^c^

^#^The values indicated with in the column by the same letters are not significantly different at 0.05 level of probability by DMRT.

### Guava leaf nutrient content

The concentrations of total nitrogen, phosphorus, potassium and micronutrients (Mn, Zn and Fe) are presented in Table 8. In the case of nitrogen, potassium, Mn, Zn and Fe the maximum value was recorded with the application of mulch @ 3.0 kg m^−2^. In the case of total phosphorus, all the mulching treatments resulted in a significant increase over the control. There was an overall improvement of 22.31% in total nitrogen, 25.28% in total phosphorus, 73.76% in total potassium, 43.00% in total iron, 29.48% in total zinc and 54.26% in total manganese in the mulched treatments over the control.

**Table 8:** Effect of mulching of *Tephrosia* biomass on guava leaf nutrient content.

Treatments	Total nitrogen (%)	Total phosphorus (%)	Total potassium (%)	Fe (ppm)	Zn (ppm)	Mn (ppm)
T_1_ (3.0 kg dry biomass m^−2^)	1.60 ± 0.06^a^	0.37 ± 0.02^a^	2.28 ± 0.06^a^	150.30 ± 5.07^a^	36.98 ± 2.09^a^	166.00 ± 14.87^a^
T_2_ (2.0 kg dry biomass m^−2^)	1.38 ± 0.04^b^	0.37 ± 0.02^a^	1.74 ± 0.08^b^	115.80 ± 3.78^b^	30.40 ± 1.74^b^	121.40 ± 7.42^b^
T_3_ (1.0 kg dry biomass m^−2^)	1.35 ± 0.03^b^	0.35 ± 0.03^a^	1.61 ± 0.11^b^	102.60 ± 4.78^c^	27.40 ± 2.35^bc^	150.40 ± 5.03^a^
T_4_(No mulching)	1.18 ± 0.06^c^	0.29 ± 0.01^b^	1.08 ± 0.15^c^	87.20 ± 4.49^d^	24.40 ± 0.77^c^	94.60 ± 4.66^c^

^#^The values indicated with in the column by the same letters are not significantly different at 0.05 level of probability by DMRT.

### Phenological behaviour of guava plants (days)

During the study period, all the trees exhibited a consistent pattern of sequential phenophases. The amount of mulch applied had a significant (*P* ≤ 0.05) effect on the duration of the phenological stages (Table 9). Application of mulch @ 3.0 kg m^−2^ resulted in a reduction in the duration of winter bud stage and leaves development stage, while there was a significant increase in the duration of bud swelling stage and appearance of flower bud stage. Plants with 3.0 kg biomass m^−2^ of mulch had the earliest bud swelling (75.36 days), in contrast to non-mulched plants, which had the longest duration of bud dormancy (104.53 days). The duration of bud swelling stage was recorded to be highest with 3.0 kg biomass m^−2^ (24.54 days) while all other treatments were at par with control. The maximum duration of appearance of flower bud was 2.46 days in the treatment consisting of the application of biomass @ 3.0 kg m^−2^ while all other treatments were at par with control.

**Table 9:** Effect of biomass mulching of *Tephrosia* on duration of phenological stages in guava.

Phenology (days)	Winter bud stage (00)	Bud swelling stage (01)	Leaves completely developed stage (19)	Appearance of flower bud stage (51)
T_1_ (3.0 kg dry biomass m^−2^)	75.36 ± 5.26^c^	24.54 ± 1.89^a^	10.94 ± 0.44^b^	2.46 ± 0.07^a^
T_2_ (2.0 kg dry biomass m^−2^)	86.53 ± 1.02^b^	19.74 ± 0.53^b^	10.76 ± 0.28^b^	1.90 ± 0.27^b^
T_3_ (1.0 kg dry biomass m^−2^)	92.46 ± 1.94^b^	18.08 ± 0.43^b^	12.46 ± 0.47^a^	1.62 ± 0.11^b^
T_4_ (No mulching)	104.53 ± 1.08^a^	17.80 ± 1.10^b^	11.44 ± 0.29^a^	1.80 ± 0.07^b^

^#^The values indicated with in the column by the same letters are not significantly different at 0.05 level of probability by DMRT.

### Guava plant growth, fruit and leaf parameters

The guava plants treated with varying quantities of *Tephrosia* mulch showed significant variation in terms of plant height (m), trunk diameter (cm), fruit yield (kg tree^−1^ and t ha^−1^), fruit weight (g), number of leaves per shoot, specific leaf area (cm^2^ g^−1^), total chlorophyll content (mg g^−1^ fresh weight) and total anthocyanin content in leaves (mg 100 g^−1^) (Table 10). However, it was interesting to note the reduction in leaf anthocyanin content in the order T_4_ > T_3_ > T_2_ > T_1_. The highest levels of other parameters were found in the case of the treatment with biomass mulching @ 3.0 kg m^−2^. There was an increase of 53.6% in the plant height and 50.47% in diameter.

**Table 10:** Effect of *Tephrosia* biomass mulching on plant growth, fruit and leaf parameters of guava.

Treatments	Plant height (m)	Trunk diameter (cm)	Yield per tree (kgtree^−1^) (winter crop 2021)	Fruit weight (kg)	Leaves per shoot	Specific leaf area (cm^2^ g^−1^)	Total chlorophyll	Total anthocyanin (mg100g^−1^)
T_1_ (3.0 kg dry biomass m^−2^)	2.95 ± 0.16^a^	9.48 ± 0.48^a^	4.04 ± 0.12^a^	111.34 ± 2.70^a^	56.04 ± 2.21^a^	109.13 ± 2.69^a^	1.68 ± 0.04^a^	7.34 ± 0.21^d^
T_2_ (2.0 kg dry biomass m^−2^)	2.53 ± 0.09^b^	8.01 ± 0.47^ab^	3.01 ± 0.20^b^	105.06 ± 5.13^ab^	47.88 ± 2.75^b^	103.79 ± 1.45^ab^	1.65 ± 0.03^a^	18.12 ± 0.32^c^
T_3_ (1.0 kg dry biomass m^−2^)	2.27 ± 0.05^b^	7.38 ± 0.84^b^	2.18 ± 0.28^bc^	104.98 ± 4.13^ab^	36.52 ± 1.94^c^	96.20 ± 2.74^bc^	1.39 ± 0.02^b^	35.27 ± 0.59^b^
T_4_ (No mulching)	1.92 ± 0.04^c^	6.30 ± 0.27^b^	2.84 ± 0.25^c^	100.30 ± 4.61^b^	15.16 ± 1.52^d^	91.98 ± 2.69^c^	1.37 ± 0.04^b^	45.45 ± 0.95^a^

^#^The values indicated with in the column by the same letters are not significantly different at 0.05 level of probability by DMRT.

### *Tephrosia* litter decomposition pattern and carbon content

**Figure 4:**
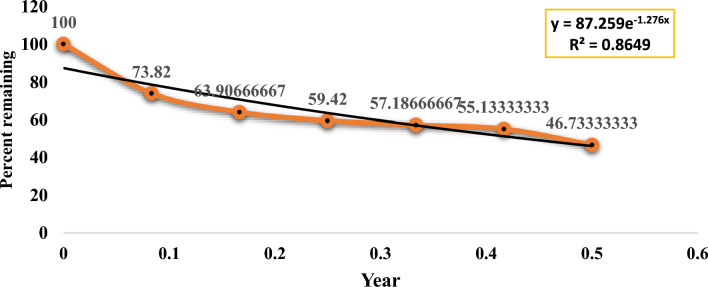
*Tephrosia* litter decomposition pattern in guava (*Psidium guajava* L.) orchard from November 2021 to May 2022.

**Figure 5:**
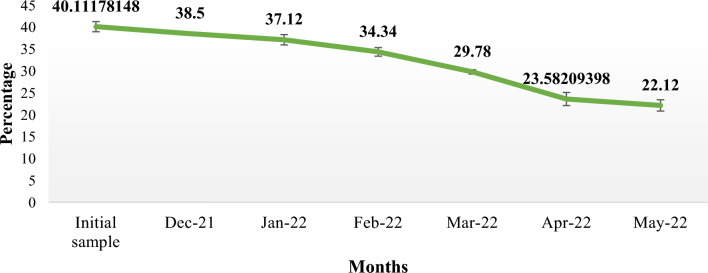
Carbon% (mean ± SE) of *Tephrosia* during litter decomposition study in guava (*Psidium guajava* L.) orchard from November 2021 to May 2022. Error bars are included to represent the standard error (SE) around the mean values.

Dry matter loss is a measure of the breakdown of litter. By the end of the sixth month (December 2021 to May 2022), 53.26% of the dry matter in the *Tephrosia* leaf litter had broken down (Fig. 4) and only 46.33% leaf litter left. *Tephrosia*'s yearly decomposition constant (k) was 1.276 (Table 11). The T_50_ (decay of 50% of biomass) and T_99_ (decay of 99% of biomass) are, 0.54 and 3.91 years, respectively. The *Tephrosia* leaf had an initial carbon content of 40.11 ± 1.15%. After six months, the *Tephrosia*'s carbon content progressively dropped (Fig. 5). There is a pattern of alternately slow and fast degradation for the carbon content. *Tephrosia* leaves lost carbon content at the following rates in December, January, February, March, April, and May: 38.50 ± 0.33%, 37.12 ± 1.17%, 34.34 ± 0.97%, 29.78 ± 0.50%, 23.58 ± 1.48%, and 22.12 ± 1.28%, respectively (Table 12).

**Table 11:** Litter decomposition constant (k), decay of 50% and 99% of biomass of *Tephrosia candida* under guava orchard.

Litter decomposition	R^2^	K	T_50_	T_99_
*Tephrosia candida*	0.8649	1.276	0.543103	3.918495

**Table 12:** Carbon% (mean ± SE) of *Tephrosia* during litter decomposition study on guava orchard from November 2021 to May 2022.

Months	Carbon% (mean ± SE)
Initial sample	40.11 ± 1.15
Dec-21	38.50 ± 0.33
Jan-22	37.12 ± 1.17
Feb-22	34.34 ± 0.97
Mar-22	29.78 ± 0.50
Apr-22	23.58 ± 1.48
May-22	22.12 ± 1.28

## Discussion

### Soil moisture dynamics

The *Tephrosia* biomass consistently maintained soil moisture levels, particularly during dry months and quantity of biomass retained was directly proportional to the amount of moisture retention. In a recent study, Das^6^ highlighted the beneficial effects of *Tephrosia candida* in retaining soil moisture during drier parts of the year in bael orchards. Hernández-Aranda^10^ also demonstrated the effectiveness of similar mulching techniques, showing a dual advantage in moisture retention and weed suppression, while also promoting microbial growth and enhancing soil structure. Moreover, Fuchs and Hadas^11^, found that a thicker mulching layer significantly increases water storage capacity, reducing runoff and enhancing resistance to water vapour, thus the quantity of mulch applied improved water retention.

### Soil electrical conductivity and soil nutrients

The study clearly indicated improvement in the soil chemical properties with mulching of *Tephrosia* biomass. The increment in soil electrical conductivity (EC) can be attributed to increased soil moisture^12^, nitrate nitrogen, or mineralizable nitrogen^13^ in the present study. When *Tephrosia* biomass is broken down, soil microorganisms break down organic compounds into simpler forms. This decomposition process releases soluble ions like potassium (K), calcium (Ca), magnesium (Mg), and sodium (Na) into the soil solution, thereby increasing ion concentration and subsequently raising electrical conductivity (EC). Additionally, as *Tephrosia* mulch decomposes, soluble ions can leach into the soil profile through rainfall. This leaching process transports dissolved ions from the mulch layer into the soil, contributing to higher ion concentration in the soil solution and consequently elevating EC levels. The quantity of mulch applied is directly related to EC, as higher mulch quantities release more ions due to processes like mineralization, immobilization, fixation, and ion release^14^. Higher EC was reported in the 0–0.15 m soil due to the prevention of leaching loss by the mulches. Mulch materials, such as *Tephrosia* mulch, create a physical barrier on the soil surface^15^. This barrier can inhibit or reduce the downward movement of water through the soil profile. As a result, water percolation and leaching of soluble ions are impeded or slowed down within the soil, particularly in the upper soil layers where the mulch is applied. By preventing or minimizing leaching losses, the mulch layer retains soluble ions, organic matter, and nutrients within the upper soil layers (0–15 cm). As water infiltrates into the soil, it interacts with the mulch layer and causes slower movement through the soil profile. This slower movement allows for greater retention of dissolved ions within the soil matrix, leading to higher concentrations of ions, nutrients and increased EC in the upper soil layers. According to Das^6^, *Tephrosia* biomass contains significant amounts of nitrogen (2.94%), phosphorus (0.16%), and potassium (2.94%). Calculations based on this data reveal that different rates of mulch application (3.0 kg m^−2^, 2.0 kg m^−2^, and 1.0 kg m^−2^) result in varying quantities of nutrients per m^2^, including nitrogen, phosphorus, and potassium. The addition of these nutrients to the plant basin through mulching materials is associated with an increase in nutrient content in the plant basin^9^. Organic mulch application plays a crucial role in reducing phosphorus movement and fixation in the soil. During decomposition, organic acids are released, which help minimize phosphorus fixation with aluminum^16^. This process contributes to the higher phosphorus content observed in the plant basin. The study observed a higher potassium content due to several factors. Firstly, organic mulch application reduces leaching, thus retaining potassium in the soil^17^. Additionally, increased soil moisture and electrical conductivity, along with organic acid release during biomass decomposition, facilitate the movement of potassium from plant tissues to the soil. Organic acids released during decomposition also form chelates with micronutrients, increasing their availability in the soil and to plants^18^. Plants more easily absorb these chelates, which are more stable than free micronutrients. Furthermore, the study suggests a net increment in soil nutrient levels in the upper depth (0–15 cm) compared to the lower depth (15–30 cm). This finding indicates that *Tephrosia* biomass improves nutrient availability through biological mineralization and by reducing runoff in the orchard^19^. These results align with previous studies, emphasizing the effectiveness of *Tephrosia* candida in improving soil nutrients, with soil nutrient availability being closely related to mulch quantity Fang^20^.

Thus, the application of *Tephrosia* biomass as mulch enriches the soil with essential nutrients, particularly nitrogen, phosphorus, and potassium. This process is facilitated by the release of organic acids during biomass decomposition, which minimizes nutrient fixation and enhances nutrient availability in the soil. Moreover, the quantity of mulch applied influences the degree of nutrient enrichment in the soil, with higher mulch quantities leading to greater nutrient availability.

### Soil organic carbon and its fractions

Soil with high organic matter content promote microbial proliferation, nutrient availability and soil structure^21^. Intercultural operations have been shown to reduce the soil carbon content by 30–50%^22^. Application of *Tephrosia* in the guava orchard helped to improve soil organic carbon and its fractions. The higher C_toc_ and C_soc_ in the surface soil might be due to the addition of biomass directly into the surface soil and lack of biological nutrient recycling in the deeper layers^23^, and this might contribute towards long term carbon sequestration. Treatments with *T*ephrosia made organic fractions better, such as highly labile, labile, and non-labile fractions. This improved crop productivity and nutrient cycling. The reater labile pool of organic carbon in the present study may be due to the higher C_mbc_, soil dehydrogenase, and soil fluoresceine diacetate activity supplied by readily decomposable carbon. *Tephrosia* litter had higher levels of ash, polyphenol, and lignin that can lead to organic carbon resistance and addition to the recalcitrant pool of soil organic carbon^24^. Therefore, the increase in non-labile organic carbon in the *Tephrosia* treated soil may be due to the quality and quantity of leaf litter. Additionally, our present study also showed a 40% carbon content in *Tephrosia candida* leaf litter, which will apparently contribute to the addition of 1.2 kg carbon per m^2^. Although *Tephrosia* litter has a higher lignin content, this contributes to its resistance to decomposition and addition to the recalcitrant pool of soil organic carbon^25^. But results are showing higher content of very labile and labile carbon fractions compared to less labile and non-labile fractions which can be explained by several factors. Firstly, *Tephrosia* litter contains a mix of labile and recalcitrant compounds. While lignin contributes to the recalcitrant nature of organic matter^25^, *Tephrosia* litter may still contain other components that are more readily decomposable, such as cellulose, hemicellulose soluble and sugars etc. These labile components can contribute to the soil's labile carbon fractions. Secondly, the initial decomposition of *Tephrosia* litter releases a significant amount of labile carbon into the soil. This influx of fresh organic matter can stimulate microbial activity, leading to faster decomposition of labile carbon fractions^26^ and higher observed values in soil analyses. Additionally, the dynamics of carbon turnover in soil involve complex interactions between organic matter inputs, microbial activity, and soil properties. Labile carbon fractions represent organic matter that is readily decomposable and can be quickly consumed by microorganisms, leading to rapid turnover^25^. When there is a lot of *Tephrosia* litter, microbes start to work, which helps break down the labile carbon fractions even more. It's important to note that the observed values of labile carbon fractions may depend on the timing and method of soil sampling, as well as the specific environmental conditions. Over time, the contributions of less labile and non-labile carbon fractions to soil organic carbon may become more apparent as decomposition progresses. Also, the *Tephrosia* biomass exhibits a C:N ratio of 13.7:1, indicating a carbon content of 40.1% and a nitrogen content of 2.9% in the leaf litter. As a result, this ratio suggests a condition that favors net mineralization. Given the aerobic nature of upland areas, fungal populations are expected to thrive, contributing significantly to the degradation of lignin^27^. Therefore, the direct application of *Tephrosia* as mulch has contributed to the increment in soil organic carbon.

### Soil biological properties

C_mbc_ generally accounts for 1–5% of C_toc_^23^ and microbial adaptation or community changes may increase carbon use efficiency^28^. *Tephrosia* mulching increased C_mbc_ due to the supply of oxidizable or mineralizable carbon, nutrients as substrates and created favourable microclimate for growth and reproduction by maintaining favourable rhizospheric microclimate. The lower value in the control may be attributed to the unfavourable rhizospheric environment, which leads to the leaching of available nutrients and increases the loss of carbon in the biomass^28^.

DHA measurement shows microbial oxidation^29^, and high dehydrogenase activity is a valid soil microbial activity metric affected by temperature, micro-biota, moisture, vegetation, and aeration^30^. Mulching with *Tephrosia* biomass reliably improved soil dehydrogenase activity over a 6-month period. Long-term effects are associated with increased oxidizable organic carbon, substrate availability (including soil nutrients and rhizospheric moisture), and soil temperature^30^. This could explain the peak in the activity of dehydrogenase during the month of April 2022 in the present investigation.

Similarly, mulching with *Tephrosia* biomass at 3.0 kg m^−2^ enhanced FDA activity. Singh^31^ and Kumawat^32^ showed similar increases in soil FDA activity in the context of residue management. A rapid rise in FDA activity was found with mulch decomposition, similar to Tang^33^, who reported a 60–120% increase in FDA activity from mulched treatments to controls. The monthly fluctuation in enzyme activity is affected by soil temperature, microbiota, moisture, vegetation, and aeration^30^. The increased FDA activity in our study implies greater microbiological activity in the mulched soil, improved biological stability of the mulched materials and improved plant nutrient availability. The decline in dehydrogenase and fluorescein diacetate (FDA) activities during the initial months up to January 2022 mainly due to reduced microbial decomposition of the newly applied mulch, because of the higher carbon-to-nitrogen (C:N) ratio, combined with lower temperatures, which collectively hinder microbial decomposition processes^34^.

### Guava leaf nutrient content

Mulching prevents root shrinkage, maintains optimal soil microclimates and soil temperature there by improves the absorption of N, P, K and micronutrients^35^. The increase in leaf nutrient content in the present study can be attributed to synergistic effect of applied nutrients^36^ which might contribute towards more absorption and translocation. As the biomass mulching enriches the soil humus, which was the source of easily available nutrients for plant absorption^37^. Fang^20^ reported that mulching with fresh grass at 7.5 kg m^−3^ resulted in increased leaf nutrient levels of popular trees, including total nitrogen, available nitrogen, available phosphorus and available potassium, compared to mulching at 5.0 kg m^−3^ and 2.5 kg m^−3^ biomass, which is in agreement with our study.

### Phenological behaviour of guava

The phenological behaviour of guava has been reported to be affected by climatic conditions such as temperature^38^, rainfall^2^, and the initial nutrient content of the soil^38^. The hastening of bud swelling by 29.17 days and a longer duration of bud swelling and appearance of the flower buds with respect to 3.0 kg m^−2^ of biomass mulching compared to non-mulching might be due to the increase in nutrient in the leaf and soil. The duration of ‘leaves completely developed stage’ was reduced by 0.5 days with mulching @ 3.0 kg m^−2^ compared to no mulching which can be attributed to the influence of degree-day accumulation and lower temperatures during vegetative flushing.

### Guava plant growth and leaf parameters

Increased guava plant growth and fruit yield with biomass mulching have been reported by several researchers^39^. The improvements in guava plant growth parameters with *Tephrosia* biomass mulching can be attributed to the improvement in soil fertility, as evidenced by increased soil nutrient and soil moisture regime. Das^6^ also reported a better tree height, trunk diameter under *Tephrosia* treatments under bael orchard. The cumulative effects of previous years' biomass mulching are responsible for the substantial enhancements in plant growth parameters in the study. Shukla^40^ has reported an increment in the fruit weight and yield of guava by use of organic fertilizers and bio fertilizers. Fang^20^ reported that mulching with fresh grass at 7.5 kg m^−3^ resulted in increased biomass production of popular trees (leaves, stems, branches, and roots) compared to mulching at 5.0 kg m^−3^ and 2.5 kg m^−3^ biomass, which is in agreement with our study. Zhang^41^ has reported that increasing soil moisture promotes nitrogen uptake in plants by improving yield. The increase in photosynthesis resulting from an increase in the number of leaves and total chlorophyll content might have contributed towards the increase in fruit weight and fruit yield. The higher leaf number recorded in the present study can be attributed to the increase in macro and micronutrient content in the soil due to biomass mulching. Leaf anthocyanin pigmentation is an indicator of low temperature stress^42^. Numerous guava genotypes commonly exhibit this phenomenon. Interestingly, present study revealed that mulching played a significant role in reduction in leaf anthocyanin content which might have contributed towards shortening of the duration of the winter bud stage in our phenological study.

### Pattern in litter decomposition study

Initial litter quality, abiotic factors, site fertility, and shifting patterns of litter microorganisms all influence the pattern of litter decomposition^43^. The litter decomposition pattern of *Tephrosia candida* biomass followed a rapid decomposition for the initial two months and then followed a slow decomposition pattern approaching T_99%_ in 3.9 years (Fig. 4). In the current research the T_99%_ values were greater than those found in the study by Rutunga^44^, Das^6^ and Ghosh and Tripathi^45^. The locations with higher deposition rates have higher T_95_% values^46^, this may be probably due to the presence of larger quantity of organic matter. The slower decomposition in the present study may also due to complex molecules like lignin, cellulose, and tannin, leading to the formation of recalcitrant organic carbon^23,47^ eventually improving soil fertility^6^. The initial leaf litter reaches its maximum decomposition limit when 20–30% of it transforms into humus. After decomposition, leaf litter forms humus and enhances numerous soil qualities. The rapid decomposition of initial leaf litter was due to the leaching loss of some of the easily soluble nutrients and non-lignified carbohydrates^23^ coupled with rainfall during the months of November 2021 and December 2021 (Fig. 1). After six months of our investigation, there had been a mass loss of 53.67% in *Tephrosia* leaf litter. Similar results were noted by Naik^23^, who found that mango leaves in hot, humid conditions lost 40% of mass by the end of 5 months.

The carbon content also followed a rapid and slow decomposition pattern (Fig. 5). This was also supported by Zhang^48^ that the slow decomposition could be caused by the low temperatures due to the winter season. The carbon content in the initial leaf litter was found to be 40.11 ± 1.15% in our study. According to Wapongnungsang and Tripathi^43^, the carbon content of *T. candida* leaves cultivated in various fallows for 3, 5, and 10 years was 30 ± 2.28%, 31 ± 2.25%, and 37 ± 2.53% respectively. After 6 months, 55.14% of the initial carbon mass remained, with only 44.86% having decomposed. This was supported by Wapongnungsang and Tripathi^43^ in their study, they found that only 50% of *Tephrosia candida* carbon content had decomposed by the end of the 6-month and by the end of 12-month mark, the carbon content was less than 20%. However, *Tephrosia candida* litter (leaves, twigs, and roots) contained 30–50% less carbon after a year, according to Lalramliani^47^.

## Conclusion

The study clearly demonstrated that mulching the guava plant basin with *Tephrosia* biomass at rates of 3.0 kg m^−2^ resulted in hastening of phenophases viz winter bud and leaf development, quicker bud swelling and the appearance of flower buds. Additionally, it resulted in higher trunk diameter, increased fruit yield, larger fruit size, and enhanced leaf parameters. Notably, it led to a significant reduction in leaf anthocyanin content. Furthermore, it effectively maintained higher soil moisture levels, even during dryer months, and significantly improved soil electrical conductivity, nutrient content (nitrogen, phosphorus, potassium and micronutrients), and organic carbon fractions. Not only that, but this study also showed that *Tephrosia* mulch is good for the soil's biological properties, including microbial biomass carbon, dehydrogenase activity, and soil fluorescein diacetate activity. *Tephrosia candida's* litter decomposition exhibited a distinct pattern, featuring rapid and slow decomposition phases. Consequently, adopting the technique of biological nutrient recycling using *Tephrosia candida* biomass mulching can be considered a sustainable agricultural approach to improve guava productivity and soil quality, leading to potential benefits for farmers and the environment, even in regions with low fertility.

## Material and method

### Experiment description

The study was conducted at Farming System Research Centre for Hill and Plateau region of ICAR Research Complex for Eastern Region situated in Ranchi district of Jharkhand state in India. The study site was located at coordinates 23° 16′ 50.4″ N latitude and 85° 24′ 39.4″ E longitude, with an elevation of 620 m above sea level. The site features laterite soil with a sandy loam texture. The average temperature ranged from 19.30 to 30.55 °C, while the average rainfall during the experiment was approximately 115.57 cm. The bulk density of the area pertaining to the experiment was 1.54 Mg m^−3^. The experiment was conducted in a 12-year-old guava (cv. Allahabad Safeda) orchard planted at a spacing of 1.0 m × 2.0 m accomodating 5000 plants ha^−1^. The long-term experiment was initiated in the year 2019. The treatments were imposed every year in the month of August. The results were obtained after third year during 2021–2022 periods. Biomass of *Tephrosia candida*, was used for imposing the treatments on mulching in the present experiment. The biomass of *Tephrosia* was harvested from the top 30% of the plant height and were mulched above soil in the whole plant basin covering entire experimental plot. The fresh biomass of *Tephrosia*, weighing 2.5 kg, is equivalent to 1 kg of *Tephrosia* biomass that has been dried in an oven. The experiment consisted of a total of four treatments viz. T_1_ (3.0 kg dry biomass of *Tephrosia candida* m^−2^ of plant basin), T_2_ (2.0 kg dry biomass of *Tephrosia candida* m^−2^ of plant basin), T_3_ (1 kg dry biomass of *Tephrosia candida* m^−2^ of plant basin), and T_4_ (control with no mulching). To maintain consistency in the treatments each year, all the plants were pruned by cutting off 50% of their shoot length. Additionally, mulches were evenly spread on the orchard floor with the same thickness in each treatment. The mulching procedure was conducted during the month of August for three consecutive years (2019, 2020 and 2021). The present investigation recorded the data pertaining to the effects of the treatments during the third year of experimentation. The experiment was undertaken under rainfed conditions with no additional application of water, chemical fertilizers or organic manures. Each treatment was replicated five times with five plants per replication and the trial was laid out in Randomized Block Design. The handling of plant material adheres to applicable institutional, national, and international guidelines and legislation.

### Observations recorded

#### Phenological behaviour of guava

In a separate experiment after pruning in October 2021, modified stages with different durations were identified, and each phenological stage was coded according to the BBCH scale. Twenty shoots from five randomly selected trees were tagged per replication in October 2021. Phenological data were recorded at four principal phenological stages viz. winter bud (00), bud swelling (01), leaves completely developed (19) and appearance of flower buds (51). Data on duration of each of the stage were recorded in days^49^.

#### Plant growth and leaf parameters

Among the plant growth parameters, we measured plant height (in meters) using a meter tape during the month of May, trunk diameter (cm) was recorded in May 2022, using vernier callipers. After harvest plants were pruned during December-January every year. The *Ambe bahar* (February–March) flowering was removed. Data on fruit weight and yield (kg tree^−1^) were collected during the winter season i.e. *Mrig bahar* (Oct 2021 to Dec., 2021) at colour break stage.

For the leaf parameters the samples were sampled during April 2022 from 5 randomly chosen shoots per plant. Leaves for total anthocyanin, were collected during December 2021. The number of leaves per shoot was counted manually. The specific leaf area (SLA) can be expressed as follows (Eq. 1):

1 $$SLA = \frac{{{\text{Leaf area }}\left( {{\text{cm}}^{2} } \right)}}{{{\text{Leaf dry mass }}\left( {\text{g}} \right)}}$$

The total chlorophyll was measured according to Hiscox and Israelstam^50^. 0.5 g of the sample was dissolved in 10 ml DMSO, and the absorbance was measured at 645 nm and 663 nm using a spectrophotometer. The total anthocyanin was measured following the method outlined by Giusti and Wrolstad^51^, by taking 2 g of the sample extracted with ethanolic HCl and the absorbance were recorded in 535 nm. The total nitrogen was determined using the Kjeldahl method outlined by Jackson^52^. 0.5 g of the sample mixed with digestion mixture and Con. H_2_SO_4_ and is distilled by using with KMnO_4_ and NaOH to release ammonia, which was then titrated against 0.1 *N* H_2_SO_4_ to determine nitrogen content. The total phosphorus was measured using the Vanado molybdate method outlined by Jackson^52^, in which 1.0 g of the sample was digested with diacid mixture and 5 ml of the diacid extract was mixed with 5 ml vanadate molybdate solution, and the absorbance of the resulting canary yellow solution was measured after 30 min using a Spectrophotometer. The total potassium was determined by flame photometry, following Jackson^52^. In this method 1.0 g of the sample was digested with diacid mixture and the diacid extract was analyzed in the flame photometer alongside potassium standards. The total micronutrients were measured by Atomic Absorption Spectrophotometry, following the method described by Lindsay and Norvell^53^. The sample (1.0 g) was digested with diacid mixture and the diacid extract was analyzed in the Atomic Absorption Spectrophotometer against standards.

#### Soil parameters

For studying the effect of *Tephrosia* biomass mulching treatments on soil parameters, soil samples were collected at distance of 30 cm away from the trunk and from two depths viz. 0–15 cm and 15–30 cm. Soil samples were obtained from 3 to 4 representative areas for each treatment, ensuring that these locations were devoid of waterlogging, standing water, or other forms of contamination. Consistency in the monthly collection was ensured by collecting samples at a fixed time each month. In the event of rain during the scheduled collection period, sampling was postponed for a minimum of 24–48 h after the rain ceased, to allow for the drainage of excess surface water from the soil. The soil moisture content was recorded gravimetrically at monthly interval from July 2021 to June 2022 and the moisture content (%) was expressed as follows (Eq. 2):

2 $${\text{Soil Moisture percentage }} = \frac{{\left( {{\text{Mw }}-{\text{Md}}} \right) \times 100}}{{{\text{Md}}}}$$

In this Eq. 2: Mw = Fresh weight of soil; Md = Dry weight of soil.

The collected soil samples were air-dried, grinded and sieved through 2 mm sieve and analyzed for various physio-chemical parameters. For the soil chemical parameters, the electrical conductivity was analyzed using a soil-to-water ratio of 1:2.5, measured with an EC meter^52^. The available nitrogen was determined as per the procedure by Subbiah and Asija^54^ with 5.0 g of soil sample was digested with 25 ml of KMnO_4_ (0.32%) and NaOH (2.5%), and the resulting ammonia was absorbed in 20 ml of 0.5% H_3_BO_3_. The distillate was titrated against 0.0175 *N* H_2_SO_4_ to determine nitrogen content. Available phosphorus was determined using Bray's method and spectrophotometric estimation with ascorbic acid^55^. 5.0 g of the sample was treated with Bray's solution, filtered, and mixed with ascorbic acid. The absorbance was measured at 660 nm after 30 min, using a standard curve to determine available phosphorus. The exchangeable potassium was determined through Flame photometry^10^. 5.0 g of soil was extracted with 25 ml of 1 *N* Ammonium acetate and filtered. The reading was taken using a flame photometer. Available Zn, Mn, Cu and Fe estimated as per the Lindsay and Norvell^53^. To the 10 g of air-dried soil, 20 ml of 0.005 M (pH 7.3) DTPA extracting solution was added, and the filtered samples were analyzed for Zn, Cu, Mn, and Fe content using an atomic absorption spectrophotometer against standards. Different fractions of carbon were determined using Modified Walkley–Black method, as described by Chan^56^. Total soil organic carbon (C_soc_) was divided into four fractions in order of decreasing oxidizability:

- Very labile OC fraction (%): Organic C oxidizable with 12 *N* H_2_SO_4_
- Labile OC fraction (%): Difference in OC oxidizable with 18 *N* H_2_SO_4_ and 12 *N* H_2_SO_4_
- Less labile OC fraction (%): Difference in OC oxidizable with 24 *N* H_2_SO_4_ and 18 *N* H_2_SO_4_
- Non labile OC fraction (%): Residual OC after reaction with 24 *N* H_2_SO_4_ and subtracted with total organic carbon (TOC). TOC was determined following the procedure by Heanes^57^.
  $${\text{OC Mg}}/{\text{ha }} = \% {\text{ OC}} \times {\text{ depth }}\left( {{\text{cm}}} \right) \, \times {\text{ bulk density}}\left( {{\text{g cm}}^{{ - {3}}} } \right)$$

Soil microbial biomass carbon (C_mbc_) was determined using the fumigation-extraction method^58^. 10 g of soil was fumigated with ethanol-free chloroform for 24 h, then extracted with 0.5 M K_2_SO_4_. After filtration, samples were treated with conc. H_2_SO_4_ + O.2N potassium dichromate + orthophosphoric acid, followed by titration against 0.05*N* ferrous ammonium sulphate (FAS) with diphenylamine indicator to compute C_mbc_ as Mg kg^−1^ soil. For analysing soil dehydrogenase activity (DHA) and Fluorescein Diacetate activity (FDA), soil samples were collected during the first week of every month from November 2021 to April 2022. DHA was assessed using 2, 3, 5 triphenyl tetrazolium chloride (TTC) following Casida^37^. 1.0 g air-dried soil was mixed with 3% TTC and 1% glucose and incubated at 37.5 °C for 24 h, then treated with methanol. Absorbance of the clear pink supernatant was measured at 485 nm using a spectrophotometer. The FDA activity in the soil was determined following Green^59^ in which 1.0 g of soil was mixed with 50 ml sodium phosphate buffer (pH 7.6, 60 mM) and 0.5 ml FDA lipase, then incubated and the absorbance of the supernatant was measured at 490 nm, and activity was calculated using a fluorescein standard curve.

#### Litter decomposition pattern

The litter decomposition study was conducted from November 2021 to May 2022 using the litter bag technique. *Tephrosia candida* leaf litter was air-dried, packed into nylon bags, and placed above the soil. Three bags were sampled monthly to analyse weight and carbon content changes. Decay constants were determined by regression analysis using Olson's negative exponential decay model^60^ (Eq. 3):

3 $${{\text{L}} \mathord{\left/ {\vphantom {{\text{L}} {{\text{Lo}}}}} \right. \kern-0pt} {{\text{Lo}}}}{ } = {\text{ e}}^{{ - {\text{kt}}}}$$

In the Eq.(3), L = the weight remaining at time t, L_o_ = the initial weight, exp. the base of the natural logarithm, k = the decay rate coefficient and t = the time (days).

The required time for 50% (t_50_) and 99% (t_99_) decay was calculated as t_50_ = 0.693 k^−1^, t_95_ = 3 k^−1^.

The carbon content of *Tephrosia* leaf was determined using the Ignition method^61^. Biomass samples were placed in pre-weighed silica crucibles and combusted in a muffle furnace at 550 °C for 3–4 h. The white ash indicated the endpoint, and final weights were measured. Carbon content was expressed as follows (Eqs. 4 and 5):

4 $${\text{Ash }}\left( {\text{\% }} \right) = \frac{{\text{Ash weight }}}{{\text{Dry weight of the sample}}} \times 100$$

5 $${\text{Carbon }}\left( {\text{\% }} \right) = {1}00 \, {-} \, \left( {{\text{ash }}\% \, + {\text{ molecular weight of O}}_{{2}} \left( {{53}.{3}} \right){\text{ in C}}_{{6}} {\text{H}}_{{{12}}} {\text{O}}_{{6}} } \right)$$

### Statistical analysis

The experiment was laid out in Randomized Block Design with five replicates and five trees per replication. The data were subjected to DMRT analysis using OPSTAT software (CCSHAU, Hissar). Mean separation was conducted with a 5% significance level using OPSTAT software.

## Data Availability

Data will be made available on request from corresponding author.
